# E-Consent—a guide to maintain recruitment in clinical trials during the COVID-19 pandemic

**DOI:** 10.1186/s13063-022-06333-6

**Published:** 2022-05-12

**Authors:** Ricardo Almeida-Magana, Hanna Maroof, Jack Grierson, Rosie Clow, Eoin Dinneen, Tarek Al-Hammouri, Nicola Muirhead, Chris Brew-Graves, John Kelly, Greg Shaw

**Affiliations:** 1 Division of Surgery & Interventional Science, University College London, Charles Bell House, 3rd Floor, 43-45 Foley Street, London, W1W 7TY UK; 2 Department of Urology, Westmoreland Street Hospital, University College London Hospital, 16-18 Westmoreland Street, London, W1G 8PH UK; 3 NCITA Clinical Trials Unit, Division of Medicine, University College London, Charles Bell House, 2nd Floor, 43-45 Foley Street, London, W1W 7TY UK

**Keywords:** e-Consent, Consent management, Prostate-cancer, Informed consent

## Abstract

**Background:**

The COVID-19 pandemic has posed daunting challenges when conducting clinical research. Adopting new technologies such as remote electronic consent (e-Consent) can help overcome them. However, guidelines for e-Consent implementation in ongoing clinical trials are currently lacking. The NeuroSAFE PROOF trial is a randomized clinical trial evaluating the role of frozen section analysis during RARP for prostate cancer. In response to the COVID-19 crisis, recruitment was halted, and a remote e-Consent solution was designed. The aim of this paper is to describe the process of implementation, impact on recruitment rate, and patients’ experience using e-Consent.

**Methods:**

A substantial amendment of the protocol granted the creation of a remote e-Consent framework based on the REDCap environment, following the structure and content of the already approved paper consent form. Although e-Consent obviated the need for in-person meeting, there was nonetheless counselling sessions performed interactively online. This new pathway offered continuous support to patients through remote consultations. The whole process was judged to be compliant with regulatory requirements before implementation.

**Results:**

Before the first recruitment suspension, NeuroSAFE PROOF was recruiting an average of 9 patients per month. After e-Consent implementation, 63 new patients (4/month) have been enrolled despite a second lockdown, none of whom would have been recruited using the old methods given restrictions on face-to-face consultations. Patients have given positive feedback on the use of the platform. Limited troubleshooting has been required after implementation.

**Conclusion:**

Remote e-Consent-based recruitment was critical for the continuation of the NeuroSAFE PROOF trial during the COVID-19 pandemic. The described pathway complies with ethical and regulatory guidelines for informed consent, while minimizing face-to-face interactions that increase the risk of COVID-19 transmission. This guide will help researchers integrate e-Consent to ongoing or planned clinical trials while uncertainty about the course of the pandemic continues.

**Trial registration:**

NeuroSAFE PROOF trial NCT03317990. Registered on 23 October 2017. Regional Ethics Committee reference 17/LO/1978.

## Introduction

As of December 2021, over 265 million COVID-19 cases and more than 5 million associated deaths have been recorded worldwide [1]. The introduction of social distancing rules to curb the spread of the virus, staff redeployment to the intensive care unit, and prioritization of COVID-19 related research has not only resulted in a complete re-organization of hospital services but has also significantly impaired the conducting of clinical trials [2]. A study reviewing the ClinicalTrials.gov registry reported that 1052 trials were suspended, as a result of the pandemic, in 2020 [3]. Governing bodies such as the United Kingdom (UK) National Health Service (NHS) Health Research Authority and the Food and Drug Administration (FDA) have therefore issued guidelines to research teams on making appropriate changes to trial protocols [4, 5]. The vast majority of these guidelines focus on a shift from on-site to remote conducting of study services.

However, obtaining patient consent poses a unique challenge, owing to the need for two-way communication between the research team and patient to ensure understanding of the provided information and subsequent documentation of approval in the form of a written signature. Previous literature has provided substantial evidence relating to the inadequacies of the traditional consent process [6]. Remote electronic consent (e-Consent) platforms have demonstrated distinct advantages over paper-based methods while also avoiding face-to-face interaction [7].

The NeuroSAFE PROOF trial is an ongoing, prospective, single-blinded, multi-centre, randomized controlled trial that enrolled adult men undergoing robot-assisted radical prostatectomy (RARP) for non-metastatic prostate cancer. Full trial description is publicly available [8]. The primary outcome is the difference in erectile function recovery between men undergoing standard RARP (control arm) and NeuroSAFE RARP (intervention arm) at 12 months following treatment. The trial enrolled its first patient in February 2019. However, recruitment was halted from March to June 2020 and from January to March 2021 due to the COVID-19 pandemic restrictions.

In response to the crisis and in line with the Health Research Authority (HRA) COVID-19 guidance released in May of 2020 [4], the NeuroSAFE PROOF research team decided to implement a Research Electronic Data Capture (REDCap) based e-Consent strategy. REDCap is a secure, web-based platform, with an integrated e-Consent feature set that was released in March of 2018 [9]. It allows for sharing of data within and across institutions, requires user authentication, and can assign data access rights based on user role to maintain confidentiality [10].

During the development process, we noticed that guidelines surrounding the implementation of e-Consent to research protocols are currently lacking [11]. This paper provides guidance on how to implement this feature and reports on the impact these changes have made on our recruitment and patient consent process.

## Methods

### Remote e-Consent instrument development

During the first recruitment pause, the University College London (UCL) Research Ethics Committee, Trial Management Group (TMG), Trial Steering Committee, and sponsor joined in a virtual meeting to decide the future of the trial. A substantial amendment of the protocol was approved that allowed the development of remote methods for screening, e-Consent, patient follow-up, and data collection.

**Fig. 1:**
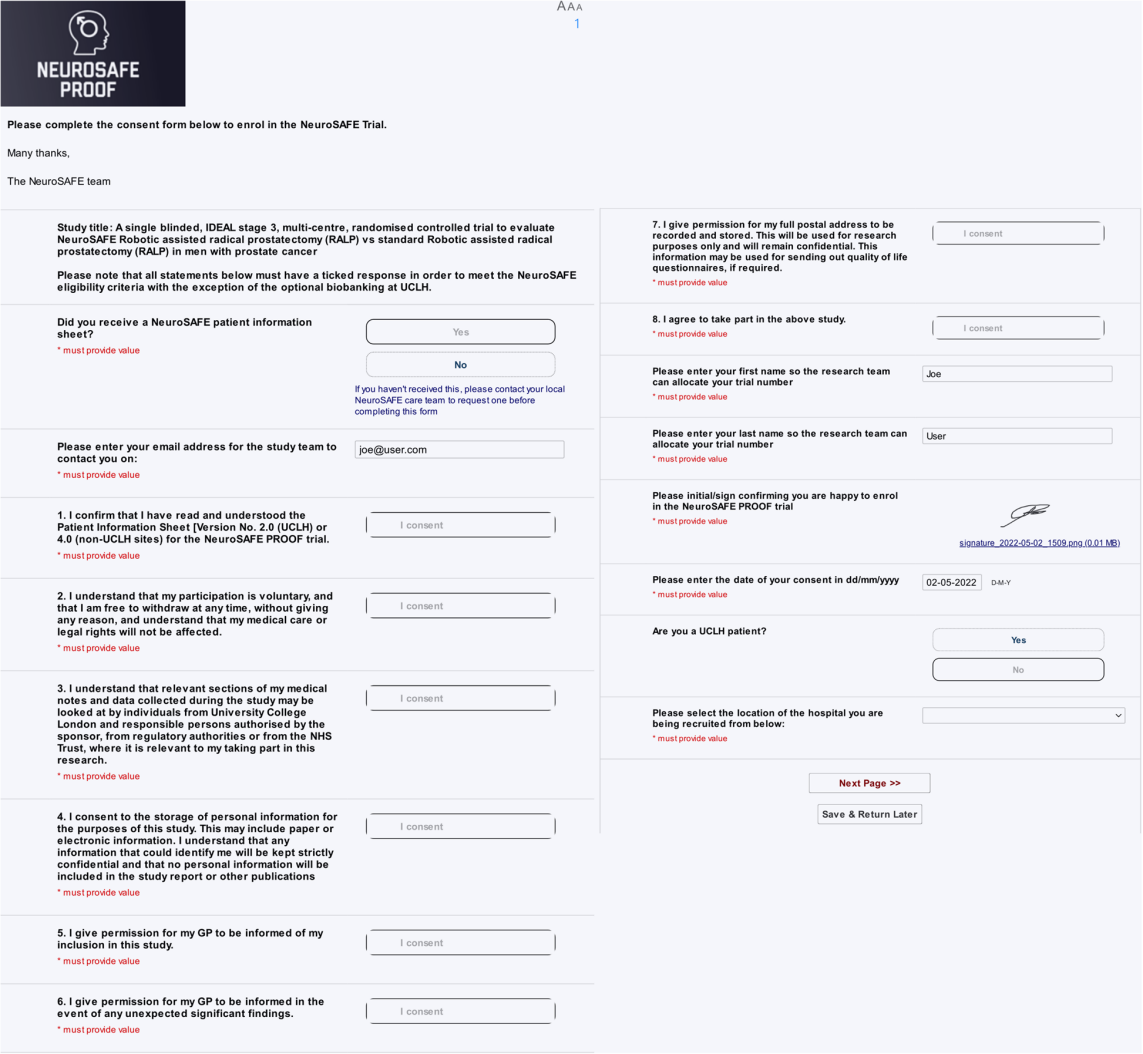
Example of a patient’s view of the e-Consent questionnaire

Our trial manager created a project for e-Consent within the REDCap environment, following the structure and content of the already approved paper consent form for NeuroSAFE Proof. The e-Consent framework works as a survey with a PDF Auto-Archiver feature (Fig. 1). Each question was added to the survey as a field with a yes/no format, except for identificatory and electronic signature fields. The latter allows the patient to sign the document using a mouse, stylus, or finger. This signature is captured and appended as a PNG image file with a timestamp.

During the enrollment process, if a patient provides an answer that disagrees with a consenting statement, the platform includes a hard stop feature, preventing ineligible patients from enrolling. The platform facilitates consent for visually impaired patients, allowing them to increase the font size as needed and features an integrated text-to-speech button.

In addition to the main trial e-Consent form, patients have the option to approve the use of the radical prostatectomy specimen obtained during surgery to be used for protocols approved by the UCL Biobanking programme for future cancer research projects.

Owing to the process collecting patient identifiable data, the platform resides within the REDCap service being hosted behind the UCL Data Safe Haven [12], which conforms to NHS Data Security & Protection Toolkit, General Data Protection Regulation, and ISO 27001 Information Security standards.

As much as possible, our team were determined to avoid missing data points. First, we instituted a system to collect patient-reported outcome measurements (PROM) via standard mail with pre-paid envelopes in accordance with scheduled clinic visits. Second, we created an approved, automatic, online platform that allowed PROMs to be completed via email link and automatically embedded into the trial database.

### Patient E-consent pathway

**Fig. 2:**
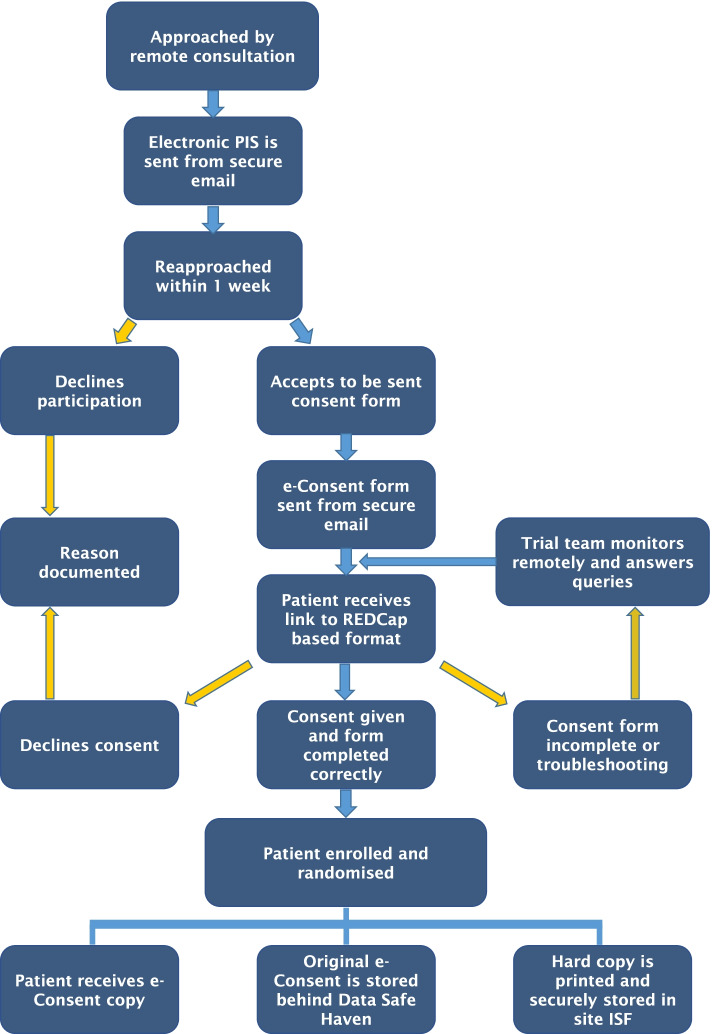
E-consent pathway diagram. Abbreviations: Patient Information Sheet (PIS), investigator site file (ISF)

Once a patient has been identified as potentially eligible for the trial in an MDT, a remote web-based virtual consultation within the normal care pathway for treatment discussion is performed by clinical staff (Fig. 2). If eligibility criteria are met, they are approached by a member of the study staff and are sent via secure NHS email a Patient Information Sheet (PIS). Within a week, they are reapproached via remote consultation. During this consultation, the patient is encouraged to ask any question while the research staff can validate patients’ understanding of the information.

If they agree, a unique e-Consent link is sent to their emails. The patient is then able to open the link on any personal electronic device and within a secure environment able to fill in the form. The absence of time pressure allowed the patient to read the consent form and a contact email is available to ask questions to the research team.

After the patient answers all questions, a certification page is added to the document, displaying a copy of the responses, allowing the patient to confirm that all the information provided is correct before final submission. Subsequently, the responses are locked. This allows an authorized member of research staff to review the file and electronically co/sign and lock the document once again. The patient is sent an electronic copy, a copy is uploaded to their electronic medical file, and a hard copy is printed and stored at the investigator site file within a locked and secure cabinet. Finally, patients who had consented and met all inclusion and exclusion criteria are randomized to either the control or intervention arm of the study.

## Results

**Fig. 3:**
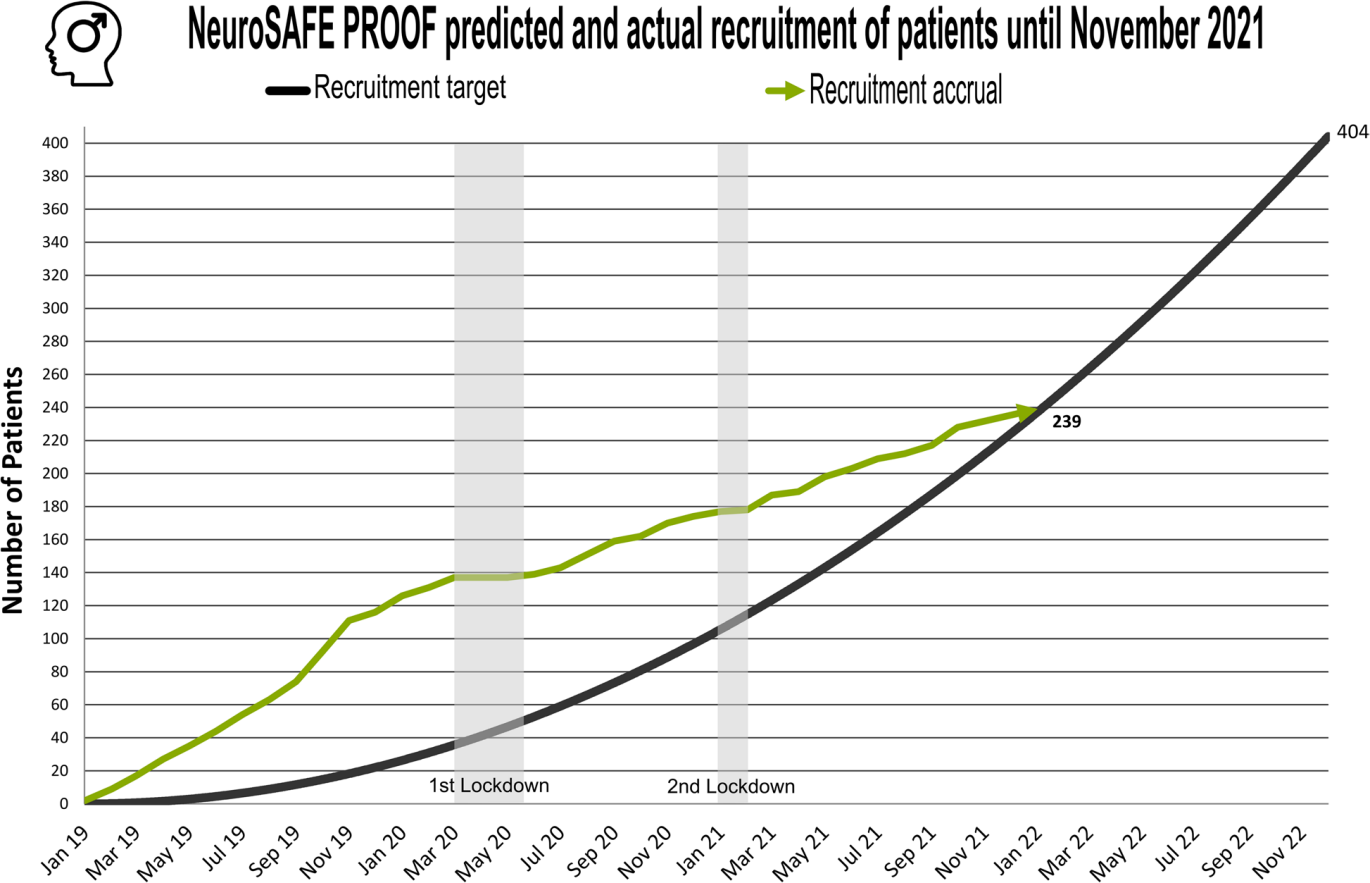
Rate of recruitment of the NeuroSAFE trial and ideal recruitment rate

At the time of institutional lockdown in the UK, NeuroSAFE PROOF had 140 men ‘on study’ at four UK sites requiring ongoing oncological surveillance or care. As per HRA governmental advice, recruitment was suspended, and all face-to-face appointments were cancelled indefinitely. The trial was placed on hold for 12 weeks. Before this pause, the trial was recruiting an average of 9 patients per month, with an increasing trend, higher than the rate required to finish the study within the proposed timeline (Fig. 3).

After implementing the remote consent and PROM collection, the NeuroSAFE PROOF study has subsequently been able to resume recruitment. We have been able to recruit 63 patients (4/month) from the main site despite a second and third lockdown. Patient acceptance to the consent process has been exceptional; only 3 patients have requested the use of a paper consent form, all three of them citing a lack of familiarity with electronic devices as the main reason for the request.

Although we have not yet returned to pre-pandemic recruitment levels, as secondary sites have just restarted normal activities, the success of our online consent system means that we can expect to reach the planned sample size of 404 patients before December 2022.

Changing the follow-up modality for postal collection of PROMs and virtual visits has allowed us to complete 188 remote outpatient follow-up consultations, with only 6% of primary outcome data missing. These results would have been completely lost if this revolutionary package had not been introduced.

## Discussion

There are three main aspects to informed consent: firstly, to engage in a comprehensive discussion with the patient about the characteristics, goals, and procedures of the study with an emphasis on voluntary participation and option to withdraw from the study at any time; secondly, to allow the patient to use this information to make an informed decision; and finally, to ensure the patients’ decision is accurately documented [13]. These objectives are all addressed via the remote web-based methods that we have detailed in this paper. At the time of writing, this was the first study to describe the use of remote e-Consent in prostate cancer research. Our data demonstrate a significant uptake in patient recruitment after remote e-Consent implementation, which has allowed the continuation of our trial while complying with the government guidelines and simultaneously minimizing infection risk for patients and researchers.

Early in the pandemic, a survey revealed that only 14% of oncology-focused European research institutions continued to enrol, with the focus being critical interventions for cancer patients [14]. Institutions were forced to implement criteria to decide which trials could continue, for example, Marcum et. al. reported that 29 of 130 active trials were cancelled at their centre [15]. A similar fate was expected to befall our trial. However, this was prevented by modification of the protocol via the described changes. Importantly, NeuroSAFE PROOF was the first trial focused on oncological surgery to reopen recruitment after a 3-month halt during the first UK lockdown. Full trial protocol will be published according to the CONSERVE recommendations [16].

Furthermore, cancer research budgets within the UK have been drastically cut over the last 2 years, as charities have struggled to fundraise, and government budgets have been diverted [17]. Strategies such as E-consent can decrease the costs associated with clinical trials and may be key when determining funding allocation in the upcoming years. This was the case for our study when trial personnel were redeployed to frontline clinical services. However, due to the straightforward nature of the platform and lack of need for paperwork, one person can consent multiple patients and simultaneously monitor their responses in real-time. The trial was therefore able to continue with minimal staffing. Furthermore, no further costs to the project budget were incurred by the implementation of the platform as it used resources already available to the sponsor (UCL).

Our trial has been transformed from in-person to almost entirely remote conduct. Patients are now only required to travel to the hospital for surgery and postoperative catheter removal. E-consent, remote collection of PROMs, and virtual consultations have allowed for this shift to occur without the quality of clinical care or research standards being compromised. The uptake of technological platforms is particularly challenging in the older cohort of patients [18]. However, our transition to online consent was relatively seamless, indicating that REDCap is a user-friendly platform and therefore a strong alternative to paper consent forms, particularly for those shielding during the pandemic.

Before the COVID-19 crisis, most e-Consent platforms relied on the use of an in-clinic electronic device [11]. Haussen et. al described the process of setting up an e-Consent platform using smartphones to communicate with legal representatives to authorize treatment for patients suffering from acute stroke [19]. This process was favoured amongst the representatives and even shortened trial enrollment time in comparison to paper consent methods. The smartphones provided adequate Internet connection and trial personnel were readily available to resolve any troubleshooting issues [20]. However, face-to-face interaction poses an increased risk of COVID-19 transmission [21] and requires setting aside time for device disinfection in between patients [22]. Not only does our platform eliminate the risk of COVID-19 transmission but also allow patients to digest provided information in their own time. Patients are then able to utilize telehealth platforms to communicate any questions or concerns surrounding the consent form, directly with the research team. In addition, such platforms will potentially broaden the patient demographic of research trials such as NeuroSAFE [23]. Patients who may not have the means to travel back and forth to the clinic, who are unable to drive due to disability, or who have caregiver responsibilities will all be able to utilize this platform to provide informed consent without leaving the confines of their homes. REDCap-based e-Consent utilizes these advantageous factors in confluence to overcome a multitude of challenges encountered with paper consent and, as a result, may lead to a definitive switch from paper-based to online consent forms in the UK, irrespective of the COVID-19 pandemic (Table 1).

**Table 1:** Advantages and disadvantages of e-Consent

*Advantages*	*Disadvantages*
Avoids physical attendance	Patients may be unfamiliar with electronic device use—increasing the digital divide and decreasing diversity of recruitment
Requires fewer human resources	Privacy concerns if not properly set-up
Requires less physical space	Could introduce selection bias towards younger patients and those with higher education
Can be deployed to any number of devices	May decrease equitable access to trials across the socioeconomic spectrum
Allows patients to answer in a safe space	Relies on patient access to electronic devices, email, and Internet connection
Can adapt to patient-specific disabilities	
Scalable	
Reduces travel-associated costs and reduces carbon footprint	
Reduces risk of contagion of infectious diseases	
Integrated hard stops prevents missing fields	
Increases traceability	
Removes postage cost	
Removes possibility of transcription errors	
Absence of time pressure	

Other e-Consent platforms have demonstrated a vast array of additional advantages that can be utilized to optimize clinical trial recruitment rates. An example is the Research Permissions Management System (RPMS), developed in South Carolina [24]. This tool not only simplified the patient recruitment process but also matched patient to trials most tailored to them. Part of the RPMS consent was a section that allowed patients to opt-in to being contacted for future research. The ability to swiftly track patients who are willing to participate in clinical trials, without having to search through paper records, will inevitably facilitate the recruitment process for future studies.

A study by Naeim et al. has also shown video-based remote e-Consent to be useful in the obtainment of consent for biospecimen collection [25]. Patients are often uneasy about the relinquishment of bodily fluids and tissue samples for research. The reasons for this may include a lack of understanding of the research process or a lack of knowledge regarding where samples will be stored. Given the importance of biospecimen studies in answering current and future translational research questions, we utilized this knowledge to incorporate an optional separate section for biospecimen collection consent within our platform, to which patients generally agreed. Lastly, physicians and researchers have also used e-Consent to gain permission to access electronic health records for both clinical care and data-sharing between institutions [26]. This will enhance collaborative research and has the advantage of being a dynamic process, in which patients can easily opt-out or request re-consent.

There are several limitations to our study. Patients may have mixed preferences towards e-Consent [27]. Elderly patients are less familiar with the use of electronic devices and tend to be more sceptical about the trustworthiness of electronic records [28]. Since the average age of our cohort is younger than in other prostate cancer studies, owing to our inclusion criteria, we acknowledge that this could be one of the reasons for our success in using this platform. In addition, our cohort was exclusively male. There is no current research to indicate whether there is a difference in gender preferences towards online consent methods. The best approach for future studies would be to integrate both remote and in-person consent of female and male patients, maximizing opportunities to observe differences in outcomes amongst patients with different backgrounds.

## Conclusions

The COVID-19 pandemic has completely changed the way clinical research is conducted and planned. Researchers must now consider social distancing restrictions while designing a protocol without compromising quality or patient safety. The implementation of a remote e-Consent pathway via REDCap was instrumental for the continuation of the NeuroSAFE PROOF trial throughout the pandemic. Both patients and regulatory bodies have accepted the use of this platform seamlessly and we have taken full advantage of the benefits of e-Consent. We hope the tools detailed in this paper will help researchers around the world make the necessary changes to ongoing studies or plan new proposals during these unprecedented times.

## Data Availability

Data sharing is not applicable to this article as no datasets were generated or analysed during the current study. The NeuroSAFE PROOF trial is ongoing, and results will be published at study completion.

## Abbreviations

e-Consent: Electronic consent
FDA: Food and Drug Administration
HRA: Health Research Authority
NHS: National Health Service
ISF: Investigator site file
PIS: Patient Information Sheet
PNG: Portable Network Graphics
PROM: Patient-reported outcome measurements
RARP: Robot-assisted radical prostatectomy
REDCap: Research Electronic Data Capture
RPMS: Research Permissions Management System
UCL: University College London
UK: United Kingdom
